# Pleuroperitoneal Leak as an Uncommon Cause of Pleural Effusion in Peritoneal Dialysis: A Case Report and Literature Review

**DOI:** 10.1155/2020/8832080

**Published:** 2020-08-31

**Authors:** Minh Huan Dang, Mathew Mathew, Rajesh Raj

**Affiliations:** Department of Nephrology, Launceston General Hospital, Launceston, Tasmania, Australia

## Abstract

Pleural effusions are frequently seen in patients on dialysis. A pleuroperitoneal leak or communication is a rare but important cause of pleural effusion in patients on peritoneal dialysis. This diagnosis can be made with a combination of biochemical tests and radiological modalities, in the absence of a gold standard diagnostic test. In addition to thoracocentesis, treatment often involves cessation of peritoneal dialysis and transition to hemodialysis. We describe a case of an 80-year-old man who presented with unilateral right-sided pleural effusion. He underwent therapeutic thoracocentesis and was subsequently diagnosed with a pleuroperitoneal leak through pleural fluid analysis. Peritoneal dialysis was ceased, and he transitioned temporarily to hemodialysis. He was subsequently treated with talc pleurodesis and successfully recommenced on peritoneal dialysis at six weeks after operation. In our report, we also review diagnostic imaging modalities, as well as advantages and disadvantages of each modality. A pleuroperitoneal leak is a rare but important complication of peritoneal dialysis and needs consideration in any patient on peritoneal dialysis presenting with unilateral pleural effusion.

## 1. Introduction

Pleural effusions are common in patients on peritoneal dialysis (PD). Determining their etiology can be challenging for clinicians since there are a wide variety of differential diagnoses. Among these, a pleuroperitoneal leak causing a pleural effusion is a rare but dramatic cause. Delays in diagnosis can lead to worsening of pleural effusions, particularly if a mistaken suspicion of fluid overload leads to the escalating use of high osmolar peritoneal dialysate fluids and volumes with the aim to achieve better ultrafiltration.

We present a case of pleuroperitoneal leak in an elderly gentleman who presented with unilateral pleural effusion after a recent change of PD mode from continuous ambulatory peritoneal dialysis (CAPD) to automated peritoneal dialysis (APD). In this report, we review the literature on this topic, explore mechanisms, and discuss treatment options.

## 2. Case Presentation

An 80-year-old gentleman on treatment with PD was admitted with increasing dyspnea and progressive reduction in ultrafiltration volumes over the previous week. His end-stage kidney disease was secondary to hypertension; he had also previously had a left nephrectomy for cancer. His comorbidities included systemic hypertension, stroke, glaucoma, prostatic hypertrophy, and osteoarthritis. He had started PD 3 months prior to admission. During the previous 2 weeks, he had switched from CAPD with daytime exchanges to nocturnal cycler-assisted automated peritoneal dialysis (APD).

**Figure 1:**
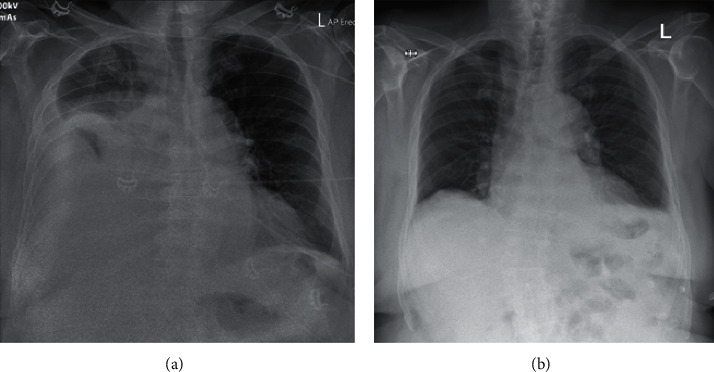
Chest X-ray on presentation (a) and after thoracentesis (b).

On initial assessment, he was tachypneic and hypoxic. The jugular venous pressure was elevated; there was also peripheral edema. On auscultation, he had reduced breath sounds over the right lower hemithorax, with dullness on percussion. His electrocardiogram (ECG) and initial blood tests were unremarkable. The chest X-ray showed a large right pleural effusion (Figure 1).

Therapeutic thoracentesis was performed. 3 litres of fluid were drained; his clinical status improved markedly. A repeat chest X-ray showed complete resolution of pleural effusion (Figure 1).

Biochemical analysis of pleural fluid showed high concentrations of sugar and creatinine, and negligible protein, consistent with peritoneal dialysate fluid (Table 1).

Treatment was changed temporarily to hemodialysis via a tunneled central venous catheter. He underwent video-assisted thoracoscopic surgery and talc pleurodesis, which he tolerated well. At the time of the procedure, most pleural fluids had been drained. There was no fresh drainage of fluid into the pleural space. Some pleural thickening was described.

4 weeks later, PD was recommenced, in CAPD mode, with small fill volumes and avoidance of nightly dwells. He subsequently tolerated CAPD without complications and was recommenced on APD. Hemodialysis was successfully discontinued.

## 3. Discussion

The incidence of pleural effusions in the dialysis population has been reported to be as high as 80% [1]. The most common causes are hypervolemia, parapneumonic effusions, and uremic pleuritis (Table 2). However, in a patient undergoing peritoneal dialysis who develops reduced ultrafiltration and pleural effusion, special consideration must be given to a possible pleuroperitoneal leak.

Pleuroperitoneal leaks are uncommon complications of peritoneal dialysis, with an estimated incidence of less than 2% [2]. The first case of a pleuroperitoneal leak was described by Edwards and Unger in 1967 [3]. Patients often present with acute dyspnea, cough, or pleuritic chest pain [4].

There are multiple predisposing mechanisms for pleuroperitoneal leaks including diaphragmatic muscular hypotonia, congenital diaphragmatic defects, pleuroperitoneal pressure gradients, and lymphatic drainage disorders [4]. A known risk factor for a pleuroperitoneal leak is polycystic kidney disease, where the high intra-abdominal pressure could lead to an increased pleuroperitoneal pressure gradient. Patients with previous episodes of peritonitis are also more likely to develop this complication, possibly related to weakened diaphragm [5].

Cases occurring within the first month of initiation of PD likely represent congenital diaphragmatic defects leading to a pleuroperitoneal communication. This leads to the development of hydrothorax, almost always on the right side once PD is started. A study of 50 patients with this complication showed 88% of cases occurring on the right side, 8% on the left, and 4% bilaterally [2]. It was suggested that the right-sided predominance occurs since the left side of the diaphragm is protected by the situation of the heart on this side [5].

There is no definitive test to confirm the diagnosis; most cases require both biochemical and radiological assessment. Biochemically, the nature of the pleural fluid is transudative [6]. Although there is no widely accepted threshold for pleural glucose concentration to diagnose the condition, a pleural glucose concentration higher than serum is highly suggestive of a pleuroperitoneal connection [7, 8].

Radiological assessment can aid diagnosis, but tests are plagued by relatively low sensitivity (Table 3). Both ultrasound and X-ray can confirm the presence of effusion, but not the source of the fluid. Peritoneal scintigraphy is considered an excellent modality to diagnose a pleuroperitoneal leak, but the sensitivity has been reported to be only 40% to 50%, and its utility in locating the site of the pleuroperitoneal communication is limited [4, 7, 9]. In a case report, Kang and Kim suggested that CT peritoneography with intraperitoneal contrast could confirm and locate the pleuroperitoneal fistula [10]. However, another study by Tang et al. showed that only 33% of patients with a pleuroperitoneal leak actually show contrast passing into the pleural cavity [11]. Documenting the appearance of methylene blue in the pleural fluid after it has been added to peritoneal dialysate has been suggested as an alternative to contrast studies; however, the test has low sensitivity, and cases of chemical peritonitis have been described [7, 11–13].

In the short term, the management of a pleuroperitoneal leak often requires discontinuation of peritoneal dialysis and a switch to hemodialysis. Thoracentesis will be required for diagnostic evaluation or symptomatic relief of large effusions. Our patient tolerated large volume thoracocentesis, with 3 L of fluid removed. Pulmonary edema has been described after large volume thoracocentesis [14]. However, our patient did not develop any complications; we suspect that this was because of rather rapid detection and treatment of his pleural effusion. Absence of adequate ultrafiltration and increasing dyspnea alerted us to the possibility early in the clinical course.

The long-term management of a pleuroperitoneal leak depends on its severity. With conservative treatment (stopping PD and continuing on hemodialysis) for six weeks to three months, the success rate is approximately 50% [15]. Alternately, for instance, in circumstances where patients have a significant residual renal function or in individuals who will be receiving a renal transplant soon, one could continue peritoneal dialysis with smaller exchange volumes, performed in Semi-Fowler's position (head and upper body inclined at 30–45 degrees to the horizontal) [16]. Surgical or chemical pleurodesis, with agents such as talc or tetracycline, are successful in ameliorating the pleuroperitoneal communication in 67–90% of cases [17, 18]. When successful, this procedure may also allow patients to return to PD therapy.

This case highlights pleuroperitoneal communication as a rare but important differential diagnosis for pleural effusions in patients on peritoneal dialysis, and especially in those with a unilateral right-sided effusion.

## Figures and Tables

**Table 1:** Serum and pleural biochemical markers.

	Serum	Pleural
Glucose (mmol/L)	8.0	12.1
Creatinine (*μ*mol/L)	527	509
Protein (g/L) (RR 60–80)	67	3
LDH (U/L)	417	60

**Table 2:** Etiology of pleural effusions.

Pleural fluid	Differential diagnoses
Transudative	Fluid overload
	Heart failure
	Pericardial diseases
	Pulmonary embolism
	Nephrotic syndrome
	Liver cirrhosis
	Pleuroperitoneal leak
	Hypothyroidism

Exudative	Pneumonia and other systemic infections
	Uremic pleuritis
	Malignancy
	Tuberculosis
	Chylothorax
	Connective tissue disorders
	Esophageal perforation
	Drug reactions

**Table 3:** Radiological modalities in diagnosing peritoneal-pleural leak.

Modality	Advantages	Disadvantages
Ultrasound	(i) Readily available	(i) Limited value in determining etiology
	(ii) Effective in confirming pleural effusion	

Plain X-ray	(i) Readily available	(i) Limited value in determining etiology
	(ii) Effective in confirming pleural effusion	

Peritoneal scintigraphy	(i) Safe and rapid way to diagnose	(i) Difficult to locate site of leak
		(ii) Requires PD-trained nurse for intraperitoneal administration of contrast

CT peritoneography	(i) Provides better resolution of anatomical details (including location and extent of extraperitoneal fluid) and can potentially locate the defect	(i) Potential nephrotoxicity with systemic absorption of contrast medium
		(ii) Requires PD-trained nurse for intraperitoneal administration of contrast

MR peritoneography	(i) Effective in diagnosing pleuroperitoneal leak	(i) Risk of nephrogenic systemic fibrosis with gadolinium (if used as contrast medium)
		(ii) More expensive and not readily available
